# Geospatial evaluation of radiologic access in Rwanda

**DOI:** 10.1186/s13244-024-01624-9

**Published:** 2024-04-08

**Authors:** Rui Han Liu, Michael Lindeborg, Isaie Ncogoza, Sabine E. Nyiraneza, Keisha J. Barrera, David A. Shaye

**Affiliations:** 1 Department of Otolaryngology-Head and Neck Surgery, Harvard Medical School, Massachusetts Eye and Ear, Boston, MA USA; 2 Department of Otolaryngology-Head and Neck Surgery, University of California San Francisco, San Francisco, CA USA; 3 University Teaching Hospital of Kigali, University of Rwanda, College of Medicine & Health Sciences, Kigali, Rwanda

**Keywords:** Diagnostic imaging, Geographic mapping, Health services accessibility, Radiology, Rwanda

## Abstract

**Background:**

Rwanda has aimed to rebuild its health care system since the Rwandan genocide against the Tutsis in 1994, though one of the challenges has been a scarcity of radiologic resources.

**Objective:**

To assess the location and accessibility of radiologic facilities in Rwanda using geospatial mapping and population-based data.

**Methods:**

A cross-sectional study was conducted in May 2023 using location and radiologic modality data provided by the Department of Radiology at the University Teaching Hospital of Kigali and the WorldPop database, a publicly available database providing open-access geospatial population data. Radiologic equipment included magnetic resonance (MR), computed tomography (CT), positron emission tomography (PET), radiotherapy, X-ray, mammography, and fluoroscopy machines. Geospatial analysis was performed using ArcGIS Pro 2.8.6 software.

**Results:**

Fifty-six radiologic facilities were identified, including 5 MR, 7 CT, 1 radiotherapy, 52 X-ray, 5 mammography, 5 fluoroscopy, and 0 PET machines. There were 0.4 MR, 0.5 CT, 0 PET, 0.1 radiotherapy, 3.9 X-ray, 0.4 mammography, and 0.4 fluoroscopy units per 1 million people.

**Conclusion:**

Rwanda is one of the countries with the lowest radiologic access in East Africa; however, there is evidence of progress, particularly in more advanced diagnostic imaging techniques such as computed tomography and magnetic resonance imaging.

**Critical relevance statement:**

This study provides a 10-year update on current radiologic resources and access in Rwanda, identifying areas of progress and ongoing scarcity, serving as a guide for future direction of growth.

**Key points:**

• As Rwanda works on rebuilding its health care system, this study provides an assessment of the current radiologic resources within the country.

• There is less than one radiologic unit for every million of the Rwandan population for every imaging modality other than X-ray.

• While radiologic access in Rwanda lags behind that of its neighbors, there has been growth focused on advanced imaging modalities and the training of human resources.

**Graphical Abstract:**

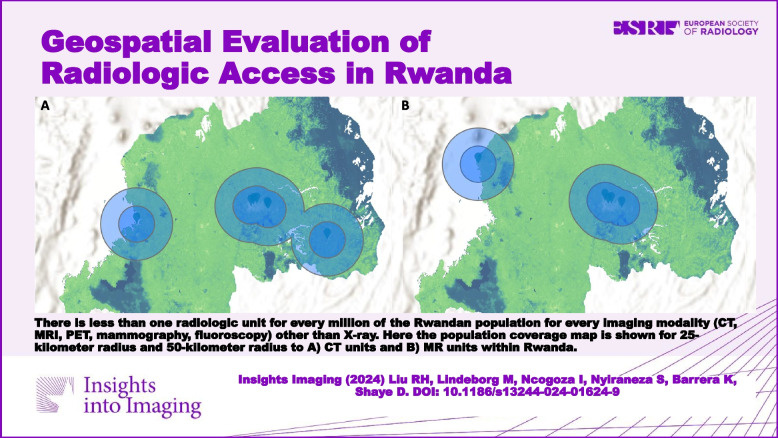

## Introduction

It is difficult to imagine modern medicine without diagnostic radiology. Radiology rapidly evolved from X-ray to magnetic resonance imaging within a span of a century and is integral to gold standard care pathways in medicine and surgery alike. However, radiology services in low- and middle-income countries (LMICs) are inadequate. While LMICs shoulder 90% of the global burden of disease, they account for only 12% of global health spending [1]. This is reflected in the severe shortage of radiologic equipment in LMICs, with a recent study from the Lancet Oncology Commission reporting less than 1 CT scanner per million population in LMICs, starkly contrasting 40 scanners per million population in high-income countries (HICs) [2]. This shortage translates to disturbing disparities in health care outcomes as many diseases, communicable (tuberculosis, human immunodeficiency virus, COVID-19) and noncommunicable (cancer, cardiovascular disorders, life-threatening emergency conditions including trauma), are reliant upon imaging for timely diagnosis and treatment [3].

One of the LMICs plagued by radiologic scarcity is Rwanda. As a sub-Saharan country in East Africa, Rwanda has a population of 14.1 million and a projected gross domestic product of 13.1 billion USD as of 2023 [4, 5]. Since the Rwandan genocide against the Tutsi and subsequent health care crisis of 1994, Rwanda has been working to rebuild its health care system. Challenges to this have been a lack of healthcare professionals, paucity of advanced infrastructure, and scarce resources [6]. It is estimated that approximately 72% of the population reside in rural areas where hospitals are sparse and services often too expensive to access [7]. However, significant progress has been made toward decentralization and accessibility, with the prioritization of training community healthcare providers, establishment of a universal health care system, and investing in health technology, including medical imaging [8].

To date, few studies have evaluated the distribution and accessibility of radiologic facilities in LMICs. In Rwanda, the most recent was a national report tabulating the radiologic resources published nearly 10 years ago [9, 10]. We aim to assess the location and accessibility of radiologic facilities in Rwanda using geospatial mapping and population-based data.

## Methods

A cross-sectional study was conducted to capture the availability of and access to radiologic equipment at a single point in time (May 2023). The Department of Radiology at the University Teaching Hospital provided the locations, radiological modalities, and public or private status of all national facilities. The radiologic modalities studied included magnetic resonance (MR), computed tomography (CT), positron emission tomography (PET), radiotherapy, X-ray, mammography, and fluoroscopy. Calculation of radiographic access was performed using the 2023 national population of 14.1 million [4].

Descriptive analysis was performed within Microsoft Excel (version 14.6.7, Microsoft Corporation, 2010) to tabulate the radiologic units per facility. Radiologic access was calculated in radiologic units per million population by dividing the total number of radiologic units by the 2023 population of Rwanda of 14.1 million.

For geospatial analysis of radiological care, GPS coordinates of public and private facilities were obtained based on information from the Department of Radiology at the University Teaching Hospital of Kigali. Hospital locations were confirmed with direct visualization using Google Earth using their corresponding latitude and longitude coordinates. Geospatial analysis was conducted using ArcGIS Pro 2.8.6. A catchment area of 25 km was chosen as the population captured within this area averaged roughly 1 million and also roughly corresponded with the sectors and districts of Rwandan units of governance. The raster dataset for population density was obtained from WorldPop data, a publicly available database that aggregates open access and high-resolution geospatial population data on population distributions, with a focus on LMICs. This study was granted exemption status by the Institutional Review Board of the University Teaching Hospital of Kigali.

## Results

**Fig. 1:**
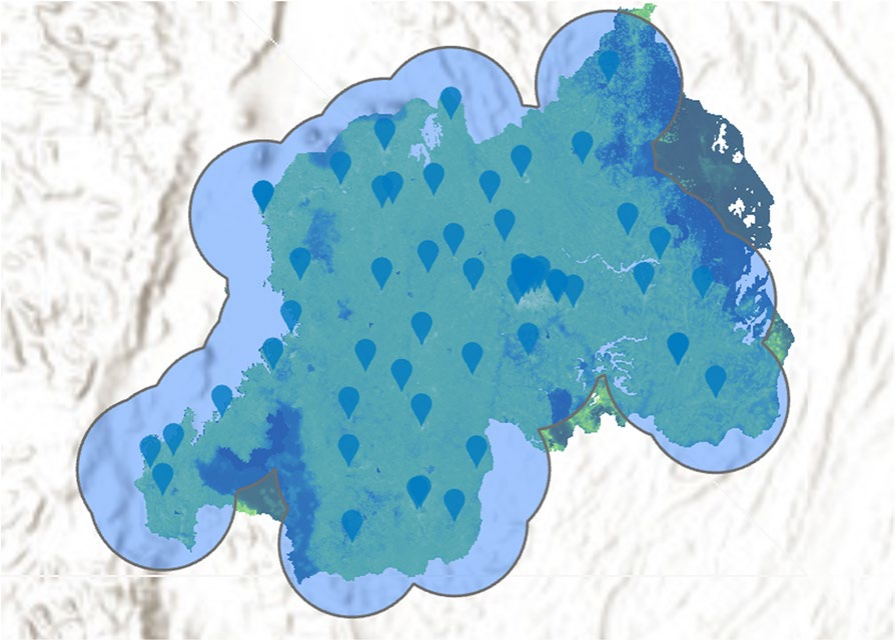
ArcGIS-generated population coverage map for 25-km radius to all radiologic facilities

**Fig. 2:**
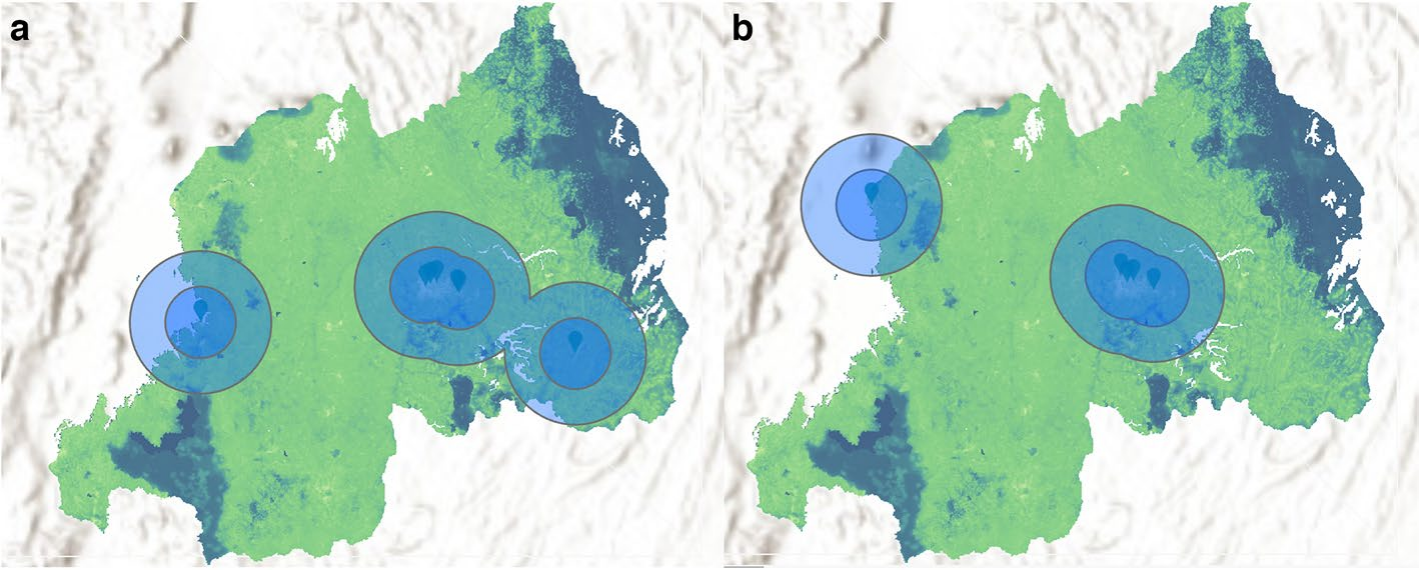
ArcGIS-generated population coverage map for 25-km radius and 50-km radius to (**a**) CT units and (**b**) MR units

A total of 56 radiologic facilities in 41 cities were identified in Rwanda, 88% (49) of which are publicly funded. The population within a 25-km radius of each of these facilities is displayed in Table 1 and ranged from 0.4 to 2.0 million people. Collectively, these facilities contain 5 MR, 7 CT, 1 radiotherapy, 52 X-ray, 5 mammography, and 5 fluoroscopy machines. There were no PET machines available in Rwanda. The breakdown of radiologic units per hospital is shown in Table 1. Notably, 13 of these radiologic facilities are in Kigali while the remaining cities had one to two facilities each. Most of the Rwandan population lives within a 25 km radius of at least one imaging modality and hospital with the exception of the more forested regions along the eastern border of Rwanda, as seen in the coverage map in Fig. 1. The map becomes much more sparsely covered when the analysis is restricted to just CT or MR units, showing that the majority of Rwanda cannot access either within 25 km (Fig. 2). Quantitatively, there were 0.4 MR, 0.5 CT, 0 PET, 0.1 radiotherapy, 3.7 X-ray, 0.4 mammography, and 0.4 fluoroscopy units per one million people. Notably, all MR units, 43% (3 out of 7) of CT units, and 40% (2 out of 5) of mammography and fluoroscopy units were privately owned.

**Table 1:** Hospitals with radiographic capability within Rwanda, population captured within a 25-km radius of each hospital, and breakdown of radiologic modalities per hospital

Hospital	City	Population within 25-km radius (in millions)	City	Radiologic modality						
				**MRI**	**CT**	**PET**	**Radiation therapy**	**X-Ray**	**Mammography**	**Fluoroscopy**
Gatonde	Busengo	1.6	Busengo	0	0	0	0	1	0	0
Kabutare	Butare	1.0	Butare	0	0	0	0	1	0	0
Butaro	Butaro Sector	0.9	Butaro Sector	0	0	0	0	1	0	0
Kirinda	Bwakira	1.0	Bwakira	0	0	0	0	1	0	0
Murunda	Colline Rugoti	0.9	Colline Rugoti	0	0	0	0	1	0	0
Gihundwe	Cyangugu	0.7	Cyangugu	0	0	0	0	1	0	0
Kibilizi	Cyarwa	0.7	Cyarwa	0	0	0	0	1	0	0
Kinihira	Gako	1.3	Gako	0	0	0	0	1	0	0
Mibilizi	Gashonga	0.6	Gashonga	0	0	0	0	1	0	0
Gakoma	Gisagara	0.7	Gisagara	0	0	0	0	1	0	0
Gisenyi District	Gisenyi	0.8	Gisenyi	1	0	0	0	0	0	0
Gisenyi	Gisenyi	0.8	Gisenyi	0	0	0	0	1	0	0
Mugonero	Gishyita	0.6	Gishyita	0	0	0	0	1	0	0
Bushenge	Gisuma	0.7	Gisuma	0	0	0	0	1	0	0
Kabgayi	Gitamara	1.2	Gitamara	0	0	0	0	1	0	0
Kibuye	Gitesi	0.7	Gitesi	0	1	0	0	1	0	0
Gitwe	Gitwe	1.1	Gitwe	0	0	0	0	1	0	0
Masaka	Kabuga	1.6	Kabuga	0	0	0	0	1	0	0
Kaduha	Karambo	0.9	Karambo	0	0	0	0	1	0	0
Rwinkwavu	Kayonza	0.6	Kayonza	0	0	0	0	1	0	0
Byumba	Kibali	1.1	Kibali	0	0	0	0	1	0	0
Kibungo	Kibungo	0.7	Kibungo	0	1	0	0	1	0	0
King Faisal	Kigali	1.7	Kigali	1	1	0	0	1	1	1
Medheal Diagnostic and Fertility Centre	Kigali	1.8	Kigali	1	1	0	0	0	0	0
Kigali Medical Imaging and Supplies Centre	Kigali	1.8	Kigali	1	0	0	0	0	0	0
Legacy	Kigali	1.7	Kigali	1	1	0	0	0	1	1
Kanombe Military/Rwanda Military	Kigali	1.7	Kigali	0	1	0	1	1	1	1
University Teaching Hospital of Kigali	Kigali	1.8	Kigali	0	1	0	0	1	1	1
Hopital la Croix du Sud	Kigali	1.8	Kigali	0	0	0	0	1	0	0
Kacyiru Police	Kigali	1.8	Kigali	0	0	0	0	1	0	0
Muhima	Kigali	1.8	Kigali	0	0	0	0	1	0	0
Kibagabaga	Kigali	1.7	Kigali	0	0	0	0	1	0	0
Nyarugenge	Kigali	1.8	Kigali	0	0	0	0	1	0	0
WIWO Specialized	Kigali	1.8	Kigali	0	0	0	0	1	0	0
University Teaching Hospital of Butare	Kigali	1.0	Kigali	0	0	0	0	1	1	1
Kibogora	Kirambo	0.5	Kirambo	0	0	0	0	1	0	0
Kirehe	Kirehe	0.5	Kirehe	0	0	0	0	1	0	0
Kigeme	Kirehe	0.8	Kirehe	0	0	0	0	1	0	0
Rutongo	Mugote	2.0	Mugote	0	0	0	0	1	0	0
Kabaya	Mukamira	1.4	Mukamira	0	0	0	0	1	0	0
Munini	Munini	0.6	Munini	0	0	0	0	1	0	0
Kiziguro	Murambi	0.8	Murambi	0	0	0	0	1	0	0
Ruli	Musasa	1.3	Musasa	0	0	0	0	1	0	0
Ngarama District	Ngarama	0.9	Ngarama	0	0	0	0	1	0	0
Muhororo	Ngororero	1.2	Ngororero	0	0	0	0	1	0	0
Nyabikenke	Nyabikenke	1.3	Nyabikenke	0	0	0	0	1	0	0
Nyagatare	Nyagatare	0.4	Nyagatare	0	0	0	0	1	0	0
Nyamata	Nyamata	1.6	Nyamata	0	0	0	0	1	0	0
Nyanza	Nyanza	1.1	Nyanza	0	0	0	0	1	0	0
Ruhango	Nyanza	1.0	Nyanza	0	0	0	0	1	0	0
Nemba	Nyarutovu	1.5	Nyarutovu	0	0	0	0	1	0	0
Ruhengeri	Ruhengeri	1.3	Ruhengeri	0	0	0	0	1	0	0
Gahini	Rukara	0.7	Rukara	0	0	0	0	1	0	0
Rwamagana	Rwamagana	0.9	Rwamagana	0	0	0	0	1	0	0
Remera-Rukoma	Taba	1.8	Taba	0	0	0	0	1	0	0
Shyira	Vunga	1.5	Vunga	0	0	0	0	1	0	0

## Discussion

This study describes the current geographic landscape of radiologic access in Rwanda. Despite most of the population living within a 25-km radius to a hospital with radiologic capability, there is significant disparity in access to certain imaging modalities, namely CT and MR, both of which are routinely used as first-line diagnostic imaging. Among the radiologic modalities included in this study, X-ray units were the most accessible, radiotherapy units were the least accessible, and PET units were inaccessible, reflective of the expense associated with purchasing, operating, and maintaining the equipment. Compared to Kenya, Tanzania, Zimbabwe, Zambia, and Uganda, Rwanda has the fewest radiologic facilities per million population [11]. Of these countries, the radiologic resources in Rwanda are most comparable to Tanzania, which has 0.4 CT units, 1.0 fluoroscopy units, 0.3 mammography units, and 0 PET units per one million people. The scarcity of Rwanda’s radiologic resources is further put into perspective once the comparative field is expanded internationally. For reference, according to Organization for Economic Co-operation and Development (OECD) countries, the United States has 38.0 MR units, 43 CT units, 70.7 mammography units, and 11.4 radiotherapy units per one million people [12–14].

Among the Rwanda population that live within a 25-km radius of a CT or MR scanner, there are further disparities to access depending on whether the equipment is located at a public or private hospital. Significantly, most of the higher-level and more expensive imaging equipment such as MR and CT scanners are private. This situation tends to pose barriers of higher cost of care, inconsistent health insurance coverage, and locations in urban centers. However, growth of private radiologic facilities may serve to reduce the burden on the public sector facilities.

There is, however, a pattern of progress in the radiologic landscape. In 2015, Rwanda had only two MR machines, both in the capital of Kigali, and five CT machines, three of which were in Kigali [10]. According to our study, there are now 3 additional MR units, one of which is in the city of Gisenyi, and 2 additional CT units, located in Kibuye and Kibungo. In contrast, there does not appear to have been any notable changes in the number of X-ray and fluoroscopy units, which perhaps alludes to an ongoing shift in the diagnostic paradigm.

Human resources are another integral piece to the radiologic landscape within Rwanda, which has been previously reported. In 2012, there were only 8 radiologists in Rwanda, with 87% (7) spending most of their time in Kigali hospitals [9]. Consequently, minimal radiologist coverage occurred at facilities outside of Kigali, where radiographic interpretation are performed predominantly by general practitioners. Much like that for radiologic equipment, there is also a growth trend for radiologists as the number of radiologists had increased from 8 in 2012 to 11 by 2014 [10]. Even so, there is still a clear deficiency of staffing for the 56 hospitals. As of 2015, there is an ongoing effort by the government to allocate resources toward a radiology residency program within Rwanda. Interestingly, in contrast to the scarcity of radiologists, there is an abundance of radiographers, averaging 10 at private hospitals and only 3 at public hospitals [9].

This study is limited by the exclusion of ultrasound as a radiologic modality due to the difficulty of obtaining accurate unit counts. However, it is known that ultrasounds are much more readily available compared to other imaging modalities in Rwanda. As a highly operator-dependent modality, ultrasound may accordingly have higher diagnostic utility in Rwanda than in more developed countries where radiologists have ready access to more advanced imaging modalities. We are therefore unable to comment on the relative diagnostic utility and necessity of the different radiologic modalities within Rwanda.

Future studies investigating the radiologic landscape in Rwanda would benefit from exploring the diagnostic utility and accuracy of the various imaging modalities, including ultrasound, as compared to more developed countries. This data would allow for realistic measures of health utility that can direct resource expansion. Additionally, data on human radiologic resources in Rwanda, including a tabulation of current technologists and radiologists, would direct further educational efforts. These studies, in conjunction with the current study, could be used to establish healthy, balanced growth of equipment and human resources.

While a comprehensive policy review is beyond the scope of this paper, the authors anticipate that improvements to the Rwandan radiologic landscape will likely take place in three areas: (1) increasing training of radiographers, (2) maximizing existing radiologic resources, and (3) expanding the repertoire of radiologic equipment. While ultrasound is the most accessible diagnostic imaging modality in Rwanda, it is arguably the most dependent on the skill of the radiographer. As radiographers gain more experience and access to subspecialty training, the diagnostic value of ultrasound is expected to increase, which may obviate the need for more advanced modalities in certain cases. Maximizing existing radiologic resources can be achieved by strengthening residency training programs and increasing the number of radiologists. Furthermore, the amount of physical radiologic equipment must be expanded, as access in Rwanda is largely limited by the scarcity of units. This ultimately will depend on the intentional allocation of health resources to equipment acquisition by the government. Overall, Rwanda has acknowledged the importance of radiologic resources in rebuilding their health care infrastructure and has made impressive strides within the past decade.

## Conclusion

Currently, Rwanda remains an East-African nation with great needs in radiologic access, with less than one radiologic unit for every million of the population for every imaging modality other than X-ray. However, there is evidence of progress in the expansion of resources with a focus on more advanced modalities such as computed tomography and magnetic resonance.

## Data Availability

Our dataset tabulating radiologic facilities and modalities is presented in Table 1 of the manuscript. The population data used in this study was taken from the open-access database WorldPop: https://hub.worldpop.org/geodata/country?iso3=RWA.

## Abbreviations

COVID-19: Coronavirus disease 2019
CT: Computed tomography
HIC: High-income countries
LMIC: Low- and middle-income countries
MR: Magnetic resonance
OECD: Organization for Economic Co-operation and Development
PET: Positron emission tomography
